# Outcomes and Predictors of Mortality for Patients with Acute Leukemia Admitted to the Intensive Care Unit

**DOI:** 10.1155/2016/3027656

**Published:** 2016-06-30

**Authors:** Alan P. Kraguljac, Danielle Croucher, Michael Christian, Narmin Ibrahimova, Vikram Kumar, Gabriella Jacob, Alex Kiss, Mark D. Minden, Sangeeta Mehta

**Affiliations:** ^1^Department of Medicine & Interdepartmental Division of Critical Care, Mount Sinai Hospital, Toronto, ON, Canada M5G 1X5; ^2^Faculty of Medicine, University of Toronto, Toronto, ON, Canada M5S 1A8; ^3^Division of Medical Oncology & Haematology, Princess Margaret Cancer Centre, Toronto, ON, Canada M5G 2M9; ^4^Department of Research Design and Biostatistics, Sunnybrook Health Sciences Centre, Toronto, ON, Canada M4N 3M5

## Abstract

*Purpose*. The objectives were to describe the management and outcomes of acute leukemia (AL) patients admitted to the ICU and to identify predictors of ICU mortality.* Methods*. Data was retrospectively collected from the medical records of all patients with AML or ALL admitted to the Mount Sinai Hospital ICU from August 2009 to December 2012.* Results*. 151 AL patients (117 AML, 34 ALL) were admitted to the ICU. Mean age was 54 (SD 15) years, median APACHE II score was 27 (IQR 22–33), and 50% were female. While in ICU, 128 (85%) patients had sepsis and 56 (37%) had ARDS. The majority of patients required invasive organ support: 94 (62%) required mechanical ventilation while 23 (15%) received renal replacement therapy. Multivariable analysis identified SOFA score (OR 1.18, 95% CI 1.01–1.38) and invasive ventilation (OR 9.64, 95% CI 3.39–27.4) as independent predictors of ICU mortality. Ninety-four (62%) patients survived to ICU discharge. Only 39% of these 94 patients discharged were alive 12 months after ICU admission.* Conclusions*. AL patients admitted to the ICU had a 62% ICU survival rate; yet only 25% of cohort patients were alive 12 months after ICU admission. Higher admission SOFA scores and invasive ventilation are independently associated with a greater risk of dying in the ICU.

## 1. Introduction

Over the past several decades, steady advances in acute lymphoblastic leukemia (ALL) and acute myeloid leukemia (AML) diagnostics, treatment regimens, and patient risk-stratification protocols have led to increased survival rates [1–5]. Nonetheless, 5-year survival rates for adult acute leukemia (AL) patients remain poor [2, 6]. Moreover, to improve AL survival rates intensive treatment regimens are used, which can lead to serious complications and admission to the intensive care unit (ICU). Outcomes for hematological malignancy (HM) patients that require ICU care, especially for those requiring mechanical ventilation or suffering from septic shock, are worse than those who avoid ICU admission [7–12]. Previously, due to the overall poor survival rates for AL patients, ICU admission for this population was largely considered futile, given concerns that critical care resources should be reserved for patients with a superior chance of survival. However, recent reports have challenged this perception by providing evidence of improving ICU survival rates amongst hematological oncology patients [4, 5, 13–16]. In order to further improve AL survival and inform the allocation of ICU resources, it is important to characterize ICU outcomes and identify predictors of patient outcome. This data may help inform clinicians, patients, and families and guide future critical care research in this vulnerable population.

In this study, we retrospectively reviewed the medical records of all AL patients admitted to Mount Sinai Hospital ICU from August 2009 to December 2012 with the following objectives: to describe patient management and outcomes and to identify variables associated with ICU and 12-month mortality.

## 2. Methods

We reviewed medical records of 151 consecutive patients with AML and ALL admitted to Mount Sinai Hospital Medical-Surgical ICU, a 16-bed teaching-ICU affiliated with the University of Toronto, from 1 August 2009 to 31 December 2012. The study was approved by the Mount Sinai Hospital and the University Health Network Research Ethics Boards, who waived the need for informed consent.

There were no defined criteria for the admission of AL patients into the ICU. ICU admission decisions were made in consultation between the attending intensivist and the patient's oncologist. Patients included in the study were identified by a medical records search for all patients ≥ 18 years admitted to ICU with the diagnosis codes of ALL or AML. For patients with multiple admissions during the study period, only data from their first admission were included in the analysis.

The majority of patients were transferred to the ICU directly from Princess Margaret Hospital (PMH), a cancer hospital, which is linked by a bridge to Mount Sinai Hospital. Patients' leukemia specific data were collected from clinical databases at PMH. These variables included leukemia classification, treatment stage, leukemia central nervous system (CNS) infiltration status, previous stem cell transplant (SCT), SCT type (allogeneic or autologous), presence of graft versus host disease (GVHD) based on clinical and pathologic data, and vital outcomes at 30, 90, 180, and 365 days after ICU admission.

All ICU related data were obtained from Mount Sinai Hospital medical records. For data collection, a standardized case report form was used to record demographics, admitting diagnosis, length of ICU stay, ICU outcome, ICU admission Acute Physiology and Chronic Health Evaluation (APACHE) II score, admission Sequential Organ Failure Assessment (SOFA) score, admission Multiple Organ Dysfunction Score (MODS), need for and duration of mechanical ventilation, need for and duration of renal replacement therapy (RRT), need for tracheostomy, presence of a confirmed infection, and administration of the following medications: vasopressors, neuromuscular blockers, steroids, sedatives, opioids, antipsychotics, antimicrobials, and chemotherapeutics. Additionally, the most abnormal laboratory results from the first 24 hours following ICU admission were collected for the following parameters: white blood cells, platelets, neutrophils, blood glucose, sodium, potassium, urea, creatinine, magnesium, bilirubin, and PaO_2_/FiO_2_ ratio. Sepsis was documented if a patient had a confirmed or probable infection (e.g., white cells in sterile body fluid, perforated viscus, radiographic evidence of pneumonia) in combination with at least 2 of the following 4 criteria within 24 hours: temperature ≥ 38°C or ≤ 36°C, heart rate ≥ 90 beats/min, respiratory rate ≥ 20 breaths/minute or the use of mechanical ventilation for acute respiratory failure, and WBC count ≥ 12,000/mm^3^ or ≤4000/mm^3^. Patients were diagnosed with septic shock if they met criteria for sepsis and received vasopressors for ≥4 hours. Acute respiratory distress syndrome (ARDS) was diagnosed according to the Berlin Definition [17].

Descriptive data are expressed as counts with percentages for categorical variables and means with standard deviation (for normally distributed data) or medians with interquartile ranges (IQR) (for nonnormally distributed data) for continuous measures. We compared characteristics of survivors and nonsurvivors at ICU discharge and 12 months after ICU admission. Chi-square (for counts > 5) and Fischer's Exact (for counts ≤ 5) tests were used to compare categorical variables, and Mann-Whitney tests were used for continuous variables. Logistic regression was used to evaluate variables associated with ICU mortality; variables entered in the model were selected a priori based on literature review and clinical expertise. Prior to analysis, the variables of interest were assessed for multicollinearity using tolerance statistics (a tolerance value < 0.4 was used as the benchmark). In the case of multicollinearity, only one member of a correlated set was retained in the multivariable model. Multivariable analysis estimates were displayed using odds ratios and their associated 95% confidence intervals. Two-tailed *P* values < 0.05 were considered statistically significant. All analyses were carried out using SAS Version 9.1 (SAS Institute, Cary, NC, USA), Microsoft Excel 2011 Version 14.3.9 (Redmond, WA, USA), or IBM SPSS Statistics Version 22.0.0 (Chicago, IL, USA).

## 3. Results

### 3.1. Cohort Characteristics

One hundred and fifty-one patients with AL were admitted to the ICU between 1 August 2009 and 31 December 2012. Mean age was 54 years (SD, 15 years), median APACHE II was 27 (IQR, 22–33), and 50% were female. The majority of patients had AML (117, 78%), while 34 (22%) had ALL; all patient subtypes are listed in Table 1. More than one-third of AL patients were admitted to ICU during the induction stage of their treatment (*N* = 55, 36%). Twenty (13%) patients had received SCT prior to admission (17 allogeneic, 3 autologous); the median time since SCT was 120 days (range 5–4215 days). Acute GVHD was diagnosed in 11 (55%) of the 20 SCT patients based on clinical and pathological features. CNS leukemic infiltration was uncommon, being confirmed in only 9 patients (6%).

Patient characteristics on the day of ICU admission are presented in Table 2. The most common admission diagnosis was sepsis (49%), followed by acute respiratory failure (31%), cardiovascular failure (10%), neurological dysfunction (5%), and gastrointestinal bleeding (3%). Notably, the vast majority of patients (128, 85%) met sepsis criteria during his/her ICU stay (at or after admission), with 58% (*N* = 87) developing septic shock. Only 38 patients (25%) had one or more infectious agent(s) confirmed microbiologically. Based on the Berlin criteria [17], ARDS was diagnosed in 56 (37%) patients. The median ICU stay was 4 days (range 2–8 days).

While in the ICU, the majority of patients required invasive organ support. Ninety-four (62%) patients required at least one method of mechanical ventilation. Noninvasive ventilation (NIV) was provided to 26 (17%) patients, of these 11 were treated with NIV alone, and 15 were intubated following NIV. In total, 82 (54%) patients required endotracheal intubation and mechanical ventilation; 12 of these 82 patients were also managed with high frequency oscillatory ventilation (HFOV). Tracheostomy was performed in the ICU in 8 (5%) patients due to prolonged mechanical ventilation. Renal replacement therapy was provided to 23 (15%) patients for acute kidney injury, for a median length of 4 days (IQR, 2–8 days). Ninety-six (64%) patients received vasopressors. Sedatives and/or opioids were provided to 104 (69%) patients (Table 2); 68 of these patients received continuous sedative and/or opioid infusion, while the remainder received only intermittent doses.

### 3.2. Outcomes and Prognostic Variables

Overall, 94 (62%) AL patients survived to ICU discharge (Table 2), with similar survival in ALL and AML patients (64% and 62%, resp.) (Table 3). Factors associated with the lowest ICU survival rates were septic shock (47% survival, *N* = 87), ARDS (48%, *N* = 56), invasive ventilation (39%, *N* = 82), HFOV (8%, *N* = 12), RRT (26%, *N* = 23), a confirmed infection (42%, *N* = 38), and vasopressors (45%, *N* = 96) or neuromuscular blockers (35%, *N* = 26) (Table 3). For patients requiring mechanical ventilation, ICU survival was lower in patients that received invasive ventilation; survival in patients treated with NIV alone (*N* = 11), NIV followed by intubation (*N* = 15), and those who were intubated without NIV treatment (*N* = 67) was 73%, 47%, and 31%, respectively (see Supplemental Table  1 of the Supplementary Material available online at http://dx.doi.org/10.1155/2016/3027656).

In bivariate analyses, nonsurvivors had significantly higher APACHE II, SOFA, and MOD scores at ICU admission compared with ICU survivors (Table 3). Furthermore, nonsurvivors had significantly higher rates of septic shock, ARDS, confirmed infections and requirement for invasive ventilation, HFOV, tracheostomy, and RRT. ICU nonsurvivors were also significantly more likely to receive vasopressors, neuromuscular blockers, and sedatives/opioids (Table 3). In the first 24 hours of admission, ICU nonsurvivors had significantly higher blood urea, creatinine, magnesium, and bilirubin levels (Supplemental Table  2). There were no differences in leukemic subtypes between ICU survivors and nonsurvivors (Supplemental Table  3).

Survival at 30, 90, and 180 days was 49%, 40%, and 34%, respectively (Table 2). Only 37 of 150 (25%) AL patients were alive 1 year after ICU admission, representing 39% of 94 patients discharged alive from ICU. One patient was excluded from analysis due to lack of vital status data. One-year mortality in patients that met septic shock criteria while in the ICU was 85%. Notably, of the 20 ICU survivors who were receiving reinduction for relapse at admission, only 3 (15%) were alive 1 year after ICU admission (Table 3). Bivariate analyses showed that 1-year nonsurvivors had significantly higher APACHE II, SOFA, and MOD scores at ICU admission (Table 3). Additionally, nonsurvivors had higher rates of septic shock and requirement for invasive ventilation, HFOV, vasopressors, and sedatives/opioids. Lastly, receiving induction therapy at the time of ICU admission was associated with higher 1-year survival.

In multivariable analysis, SOFA score at ICU admission (OR 1.18, 95% CI 1.01–1.38) and invasive ventilation (OR 9.64, 95% CI 3.39–27.4) were independently associated with death in the ICU (Table 4). Age at admission (OR 1.01, 95% CI 0.98–1.05) and septic shock (OR 4.06, 95% CI 0.97–7.50) were not identified as independent predictors of ICU mortality.

## 4. Discussion

ICU admission for cancer patients is a highly debated topic. When resources are limited, intensivists and oncologists must restrict admissions to patients with a reasonable opportunity for recovery. Studies from European centres have reported that 25–51% of cancer patients requiring ICU care are refused at admission [18, 19]. However, ICU survival rates are increasing for cancer patients and in-hospital survival rates are similar to patients with other comorbidities such as liver cirrhosis or heart disease [4, 15]. These observations suggest that additional predictors of ICU survival are required to identify cancer patients that can benefit most from ICU admission.

In this retrospective study of 151 consecutive AL patients admitted to the ICU, we found that ICU and 1-year mortality rates were 38% and 75%, respectively. These survival rates are comparable to previous studies of HM patients admitted to the ICU [5, 7, 14, 16, 20–22]. Mortality did not differ significantly between AML and ALL patients. Notably, our patient population demonstrated a high requirement for invasive therapies and lengthy ICU stay. Bivariate analyses identified several variables associated with both ICU and 1-year mortality including higher illness severity scores, septic shock, invasive ventilation, HFOV treatment, and management with opioids or vasopressors. Our multivariable analysis identified SOFA score and invasive ventilation as independent predictors of ICU mortality.

Illness severity scores and management with invasive ventilation are widely described as predictive of mortality in patients with HM [23–26]. Similar to our observations, a retrospective review on the ICU stay of 58 HM patients by Cornet et al. reported that ICU nonsurvivors had significantly higher SOFA scores than nonsurvivors on the first 4 days of ICU admission [23]. Moreover, in a study of AL patients requiring invasive ventilation, Price et al. found that each increase in SOFA score of 1 point at the time of intubation was associated with a 17% increase in mortality [24]. This observation suggests that future studies should investigate if SOFA scores from additional time points (intubation, 48 hours after admission, and postsurgical procedures) might be better predictors of survival than at admission alone. In our study, patients that received invasive ventilation were less likely to survive to ICU discharge than those avoiding intubation. ICU survival rates of 73% for patients treated with NIV alone dropped to 47% when invasive ventilation was subsequently required and to 31% for those who were intubated without NIV therapy. This observation is consistent with a study from Rabbat et al. [14] that found a 67% survival rate for HM patients initially treated with NIV and 13% for patients who were provided first-line invasive mechanical ventilation.

In contrast to other similar studies [7, 14, 26–28], we did not identify septic shock, leukemic subtype, or neutropenia as independent predictors of ICU mortality. Yet, we observed that septic shock was trending towards being a significant independent predictor of mortality. In accordance with this observation, other studies report that ICU patients with septic shock (with or without HM) have high mortality rates ranging from 20 to 30% [27]. Moreover, an observational study from Park et al. [28] reporting on outcomes of 50 HM patients admitted to the ICU with septic shock and a recent review by Azoulay et al. [4] suggest that mortality rates for HM patients with septic shock are as high as 47–60%. Thus, while outcomes of HM patients with septic shock are improving [29], it appears that this population still has a high risk of dying in the ICU. Secondly, leukemic subtype or cytogenetic risk group has also been reported as predictive of outcomes [7, 14, 22]. Importantly, Thakkar et al. found that while cytogenetic risk group is predictive of 6-month mortality, this predictive ability is similar for those HM patients with comparable cytogenetics that avoid ICU care [7]. This finding implies that leukemic subtype does not influence short-term survival. Lastly, while neutropenia has been described as an independent predictor of mortality for patients with HM [9, 26], it is no longer considered relevant [4, 5] due to overall improvements in the understanding and management of chemotherapy-related neutropenia.

Strengths of our study include the large cohort relative to many other studies investigating outcomes of HM patients admitted to the ICU [9]. Our focus on AL patients provides more tailored data towards this population than many previous reports, which commonly include all HM patients. Our study has limitations. The study was conducted in a single centre with no formal ICU admission criteria, potentially resulting in selection bias. While we collected 1-year postadmission survival status for 150 patients, quality of life (QOL) and functional status measures were unavailable. Importantly, QOL measures provide important prognostic information for intensivists and patients, as survivors may be bed-ridden or physically incapacitated. There is growing evidence that early ICU admission, even before organ failure has developed [16], may correlate with better outcomes [16, 18, 30, 31]; however we could not determine if ICU admission timing influenced survival rates in our cohort due to the retrospective nature of our report.

In conclusion, we identified SOFA score at admission and invasive ventilation as independent predictors of mortality in AL patients requiring ICU admission. Although 62% of patients survived their ICU stay, only 1 in 4 patients were alive at 1-year after admission. This observation suggests that we require additional investigations to help identify patients with HM that will benefit in the long term from ICU care.

## Supplementary Material

The supplemental material contains ICU survival rates for each ventilation strategy observed (Supplemental Table 1), bivariate analyses comparing standard laboratory test values (from the first 24 hours of ICU admission) of ICU survivors and non-survivors (Supplemental Table 2) and bivariate analysis on potential influence of leukemic subtype on ICU and 1-year survival (Supplemental Table 3).

## Figures and Tables

**Table 1:** Patient leukemia characteristics (*N* = 151).

	*N* (%)
*Leukemia type*	
Acute myeloid leukemia	118 (78)
M0	12 (8)
M1	17 (11)
M2	14 (9)
M3	1 (1)
M4	19 (13)
M5	13 (9)
Other	42 (28)
Acute lymphoblastic leukemia	33 (22)
B-ALL	26 (17)
T-ALL	5 (3)
Mixed phenotype	2 (1)

*Chemotherapy stage*	
Induction	55 (36)
Consolidation	9 (6)
Intensification phase	5 (3)
Reinduction for relapse	29 (20)
Reinduction for nonresponse	12 (8)
Not provided chemotherapy within 40 days	33 (22)
No pharmacy records	3 (2)
Other^a^	5 (3)

*Leukemia factors*	
CNS infiltration	9 (6)
SCT before ICU	20 (13)
Allogeneic	17
Autologous	3
GVHD	11 (7)

Data presented as *N* (% of total patients).

CNS = central nervous system, SCT = stem cell transplant, and GVHD = graft versus host disease.

^a^Other = maintenance therapy or conditioning regimen.

**Table 2:** Patient characteristics and outcomes (*N* = 151).

	*N* ^*∗*^
*Characteristics at admission*	
Age, years	54 (15)^Ŧ^
Female	76 (50)
APACHE II score	27 (22–33)
SOFA score	10 (8–13)
MODS	7 (6–10)
Neutropenia (neutrophils < 2.0 × 10^9^/L)	84 (74)
Thrombocytopenia (platelets < 50 × 10^9^/L)	126 (86)

*Admission diagnosis*	
Sepsis	74 (49)
Respiratory	46 (31)
Cardiovascular failure	15 (10)
Gastrointestinal bleed	3 (2)
Neurological dysfunction	8 (5)
Other	5 (3)

*Morbidity in ICU*	
Sepsis	128 (85)
Septic shock	87 (58)
Acute respiratory distress syndrome	56 (37)
Stroke	8 (5)

*Treatments in ICU*	
Mechanical ventilation	94 (62)
NIV alone	11 (7)
NIV followed by intubation	15 (10)
Invasive alone	67 (44)
Total invasive ventilation	82 (54)
Total NIV	26 (17)
High frequency oscillation	12 (8)
Tracheostomy	8 (5)
Renal replacement therapy	23 (15)
Duration of renal replacement therapy (days)	4 (2–6)
Insulin infusion	50 (33)

*Infections identified in ICU *	
Microbiologic pathogen identification	38 (25)^*∗∗*^
Bacterial	27 (18)
Viral	8 (5)
Fungal	7 (5)

*Medications administered in ICU*	
Antibiotics	144 (95)
Antifungals	105 (70)
Chemotherapy	41 (27)
Sedatives/opioids	104 (69)
Infusion +/− boluses	68 (45)
Intermittent alone	36 (24)
Vasopressors	96 (64)
Neuromuscular blockers	26 (17)
Steroids	47 (31)
Antipsychotics	24 (16)

*Outcomes*	
ICU stay, days	4 (2–8)
ICU survival	94 (62)
30-day survival	74 (49)
90-day survival	60 (40)
180-day survival	51 (34)
365-day survival	37 (25)

^*∗*^Data presented as *N* (%) for categorical variables and mean (SD)^Ŧ^ or median (IQR) for continuous measures.

^*∗∗*^Total number of bacterial, viral, and fungal infections is >38 as some patients had more than 1 pathogen identified.

APACHE = acute physiology and chronic health evaluation score, MODS = multiple organ dysfunction score, SOFA = sequential organ failure assessment score, and NIV = noninvasive ventilation.

**Table 3:** Characteristics of ICU survivors and nonsurvivors at ICU discharge and 1 year after ICU admission.

Variable	ICU discharge			1-year after ICU admission		
	Survivors	Nonsurvivors	*P* value	Survivors	Nonsurvivors	*P* value
	*N* = 94	*N* = 57		*N* = 37	*N* = 113	
*Leukemia type*						
Acute myeloid leukemia	73 (78)	45 (79)	0.85	26 (70)	91 (80)	0.19
Acute lymphoblastic leukemia	21 (22)	12 (21)	0.85	11 (30)	22 (20)	0.19

*Chemotherapy stage *						
Induction	37 (39)	18 (32)	0.34	20 (55)	34 (30)	0.008
Consolidation	6 (7)	3 (5)	1	2 (5)	7 (6)	1
Intensification phase	5 (5)	0 (0)	0.16	3 (8)	2 (2)	0.1
Reinduction for relapse	20 (21)	9 (16)	0.41	3 (8)	26 (23)	0.05
Reinduction for nonresponse	7 (8)	5 (9)	0.77	2 (5)	10 (9)	0.73
Not provided chemotherapy within 40 days	14 (15)	19 (33)	0.008	5 (14)	28 (24)	0.15
Unclear (no pharmacy records)	0 (0)	3 (5)	0.05	0 (0)	3 (3)	1
Other	5 (5)	0 (0)	0.16	2 (5)	3 (3)	0.6

*Leukemic factors*						
CNS infiltration	8 (9)	1 (2)	0.08	2 (5)	7 (6)	1
SCT before ICU	13 (14)	7 (12)	0.79	5 (14)	15 (13)	1
GVHD	6 (6)	5 (9)	0.11	4 (11)	7 (6)	1

*Characteristics*						
Age	54 (22)	57 (17)	0.42	53 (19)	57 (20)	0.12
APACHE II	25 (20–30)	30 (24–39)	0.001	26 (22–30)	28 (21–33)	0.046
SOFA	9 (6–11)	13 (10–15)	<0.001	8 (7–15)	10 (8–13)	<0.001
MODS	7 (5–9)	8 (7–11)	0.001	7 (5–9)	8 (6–10)	0.039
ICU length of stay (days)	4 (2–7)	5 (3–9)	0.3	4 (3–8)	4 (2–9)	0.71

*Morbidity in ICU*						
Sepsis	77 (82)	51 (90)	0.21	30 (81)	97 (86)	0.49
Septic shock	41 (44)	46 (81)	<0.001	13 (35)	73 (65)	0.002
ARDS	27 (29)	29 (51)	0.004	11 (30)	43 (38)	0.36
Stroke	4 (4)	4 (7)	0.48	1 (3)	7 (6)	0.68

*Treatments in ICU*						
Invasive ventilation	32 (34)	50 (88)	<0.001	10 (27)	71 (63)	<0.001
*Duration of invasive ventilation (days)*	7 (4–20)	6 (2–9)	0.07	6 (2–10)	6 (3–13)	0.33
Noninvasive ventilation (NIV)	15 (16)	11 (20)	0.6	5 (14)	21 (19)	0.5
*Duration of NIV (days)*	2 (1–3)	2 (2-3)	0.57	2 (1-2)	2 (1–3)	0.34
High frequency oscillation	1 (1)	11 (20)	<0.001	0 (0)	12 (11)	0.039
Tracheostomy	8 (9)	0 (0)	0.024	2 (5)	5 (4)	1
Renal replacement therapy (RRT)	6 (6)	17 (30)	<0.001	2 (5)	20 (18)	0.07
*Duration of RRT (days)*	6 (3–19)	4 (2–6)	0.39	3 (2–5)	6 (2–9)	0.55
Insulin infusion	26 (28)	24 (42)	0.07	10 (27)	40 (35)	0.35

*Infections identified in ICU*						
Confirmed infection^*∗*^	16 (17)	22 (39)	0.003	5 (14)	32 (28)	0.07
Bacterial	13 (14)	14 (25)	1	4 (11)	22 (20)	1
Viral	3 (3)	5 (9)	0.68	1 (3)	7 (6)	1
Fungal	2 (2)	5 (9)	0.3	0 (0)	6 (5)	0.57

*Medications administered in ICU*						
Antibiotics	92 (98)	52 (91)	0.06	36 (97)	107 (95)	1
Antifungals	65 (69)	40 (70)	0.89	23 (62)	81 (72)	0.28
Chemotherapy	28 (30)	13 (23)	0.35	14 (38)	27 (24)	0.1
Sedatives/opioids	51 (54)	53 (93)	<0.001	19 (51)	84 (74)	0.009
Vasopressors	43 (46)	53 (93)	<0.001	15 (41)	81 (72)	0.001
Neuromuscular blockers	9 (10)	17 (30)	0.001	4 (11)	21 (19)	0.27
Steroids	24 (26)	23 (40)	0.06	7 (19)	39 (35)	0.07
Antipsychotics	14 (15)	10 (18)	0.67	2 (5)	21 (19)	0.053

Data presented as *N* (%) for categorical variables and median (IQR) for continuous variables.

APACHE = acute physiology and chronic health evaluation, CNS = central nervous system, GVHD = graft versus host disease, MODS = multiple organ dysfunction score, SCT = stem cell transplant, and SOFA = sequential organ failure assessment score.

^*∗*^Total number of bacterial, viral, and fungal infections in each column may be greater than the “confirmed infection” as some patients had more than 1 infection.

**Table 4:** Multivariable analysis of independent predictors of ICU mortality.

Variable	Odds ratio estimates (95% Wald CI)	*P* value
SOFA	1.18 (1.01–1.38)	0.034
Induction stage at ICU admission	0.43 (0.16–1.17)	0.097
Relapse stage at ICU admission^*∗*^	0.49 (0.14–1.66)	0.248
Age	1.01 (0.98–1.05)	0.387
Septic shock	4.06 (0.97–7.50)	0.057
Invasive ventilation	9.64 (3.39–27.40)	<0.001

CI = confidence interval and SOFA = sequential organ failure assessment.

^*∗*^Includes only reinduction for relapse patients.

## References

[1] Azoulay É., Afessa B. (2006). The intensive care support of patients with malignancy: do everything that can be done. *Intensive Care Medicine*.

[2] Bassan R., Hoelzer D. (2011). Modern therapy of acute lymphoblastic leukemia. *Journal of Clinical Oncology*.

[3] Inaba H., Greaves M., Mullighan C. G. (2013). Acute lymphoblastic leukaemia. *The Lancet*.

[4] Azoulay E., Pène F., Darmon M. (2015). Managing critically Ill hematology patients: time to think differently. *Blood Reviews*.

[5] Van Vliet M., Verburg I. W. M., Van Den Boogaard M. (2014). Trends in admission prevalence, illness severity and survival of haematological patients treated in Dutch intensive care units. *Intensive Care Medicine*.

[6] Ferrara F., Schiffer C. A. (2013). Acute myeloid leukaemia in adults. *The Lancet*.

[7] Thakkar S. G., Fu A. Z., Sweetenham J. W. (2008). Survival and predictors of outcome in patients with acute leukemia admitted to the intensive care unit. *Cancer*.

[8] Lloyd-Thomas A. R., Wright I., Lister T. A., Hinds C. J. (1988). Prognosis of patients receiving intensive care for lifethreatening medical complications of haematological malignancy. *British Medical Journal*.

[9] McDowall K. L., Hart A. J., Cadamy A. J. (2011). The outcomes of adult patients with haematological malignancy requiring admission to the intensive care unit. *Journal of the Intensive Care Society*.

[10] Rabe C., Mey U., Paashaus M. (2004). Outcome of patients with acute myeloid leukemia and pulmonary infiltrates requiring invasive mechanical ventilation—a retrospective analysis. *Journal of Critical Care*.

[11] Tremblay L. N., Hyland R. H., Schouten B. D., Hanly P. J. (1995). Survival of acute myelogenous leukemia patients requiring intubation/ventilatory support. *Clinical and Investigative Medicine*.

[12] Sippel C., Kim Y., Wallau A., Brossart P., Schmidt-Wolf I., Walger P. (2015). AML versus ICU: outcome of septic AML patients in an intensive care setting. *Journal of Cancer Research and Clinical Oncology*.

[13] Hill Q. A. (2010). Intensify, resuscitate or palliate: decision making in the critically ill patient with haematological malignancy. *Blood Reviews*.

[14] Rabbat A., Chaoui D., Montani D. (2005). Prognosis of patients with acute myeloid leukaemia admitted to intensive care. *British Journal of Haematology*.

[15] Azoulay E., Soares M., Darmon M., Benoit D., Pastores S., Afessa B. (2011). Intensive care of the cancer patient: recent achievements and remaining challenges. *Annals of Intensive Care*.

[16] Lengliné E., Raffoux E., Lemiale V. (2012). Intensive care unit management of patients with newly diagnosed acute myeloid leukemia with no organ failure. *Leukemia & Lymphoma*.

[17] Ranieri V. M., Rubenfeld G. D., Thompson B. T. (2012). Acute respiratory distress syndrome: the Berlin Definition. *The Journal of the American Medical Association*.

[18] Azoulay E., Mokart D., Pène F. (2013). Outcomes of critically ill patients with hematologic malignancies: prospective multicenter data from France and Belgium—a groupe de recherche respiratoire en réanimation onco-hématologique study. *Journal of Clinical Oncology*.

[19] Thiéry G., Azoulay É., Darmon M. (2005). Outcome of cancer patients considered for intensive care unit admission: a hospital-wide prospective study. *Journal of Clinical Oncology*.

[20] Ferrà C., Marcos P., Misis M. (2007). Outcome and prognostic factors in patients with hematologic malignancies admitted to the intensive care unit: a single-center experience. *International Journal of Hematology*.

[21] Magid T., Haase N., Andersen J. S., Nielsen O. J., Bonde J. (2012). Intensive care of haematological patients. *Danish Medical Journal*.

[22] Jackson K., Mollee P., Morris K. (2014). Outcomes and prognostic factors for patients with acute myeloid leukemia admitted to the intensive care unit. *Leukemia & Lymphoma*.

[23] Cornet A. D., Issa A. I., van de Loosdrecht A. A., Ossenkoppele G. J., Strack van Schijndel R. J. M., Groeneveld A. B. J. (2005). Sequential organ failure predicts mortality of patients with a haematological malignancy needing intensive care. *European Journal of Haematology*.

[24] Price K. J., Cardenas-Turanzas M., Lin H., Roden L., Nigam R., Nates J. L. (2013). Prognostic indicators of mortality of mechanically ventilated patients with acute leukemia in a comprehensive cancer center. *Minerva Anestesiologica*.

[25] Bird G. T., Farquhar-Smith P., Wigmore T., Potter M., Gruber P. C. (2012). Outcomes and prognostic factors in patients with haematological malignancy admitted to a specialist cancer intensive care unit: a 5 yr study. *British Journal of Anaesthesia*.

[26] Ñamendys-Silva S. A., González-Herrera M. O., García-Guillén F. J., Texcocano-Becerra J., Herrera-Gómez A. (2013). Outcome of critically ill patients with hematological malignancies. *Annals of Hematology*.

[27] Angus D. C., van der Poll T. (2013). Severe sepsis and septic shock. *The New England Journal of Medicine*.

[28] Park H. Y., Suh G. Y., Jeon K. (2008). Outcome and prognostic factors of patients with acute leukemia admitted to the intensive care unit for septic shock. *Leukemia and Lymphoma*.

[29] Pène F., Percheron S., Lemiale V. (2008). Temporal changes in management and outcome of septic shock in patients with malignancies in the intensive care unit. *Critical Care Medicine*.

[30] Song J.-U., Suh G. Y., Park H. Y. (2012). Early intervention on the outcomes in critically ill cancer patients admitted to intensive care units. *Intensive Care Medicine*.

[31] Mokart D., Lambert J., Schnell D. (2013). Delayed intensive care unit admission is associated with increased mortality in patients with cancer with acute respiratory failure. *Leukemia and Lymphoma*.

