# Supplementary material for: Outcomes and Predictors of Mortality for Patients with Acute Leukemia Admitted to the Intensive Care Unit
Source: Can Respir J. 2016 Jun 30;2016:3027656. doi: 10.1155/2016/3027656 (PMC4944052; doi:10.1155/2016/3027656)
Supplement: Supplementary file 1 — The supplemental material contains ICU survival rates for each ventilation strategy observed (Supplemental Table 1), bivariate analyses comparing standard laboratory test values (from the first 24 hours of ICU admission) of ICU survivors and non-survivors (Supplemental Table 2) and bivariate analysis on potential influence of leukemic subtype on ICU and 1-year survival (Supplemental Table 3). [file 3027656.f1.docx]

Supplemental Table 1. Patient survival rates based on ventilation strategy

| Ventilation Strategy Provided | N (% of total patients) | Survival  N (%) |
| --- | --- | --- |
| NIV alone  NIV followed by invasive (intubation)  Invasive alone  Total Invasive Ventilation  Total Non-Invasive Ventilation  High Frequency Oscillation  Tracheostomy | 11 (7)  15 (10)  67 (44)  82 (54)  26 (17)  12 (8)  8 (5) | 8 (73)  7 (47)  22 (31)  44 (39)  14 (58)  1 (8)  8 (100) |

Supplemental Table 2. Laboratory tests at ICU admission in survivors and non-survivors

|  | **Total ICU Population (N=151)** | | **ICU Survivors**  **(N=94)** | | **ICU Non-survivors (N=57)** | |  |
| --- | --- | --- | --- | --- | --- | --- | --- |
| **Laboratory Test**  White Blood Cells (10^9^/L)  Neutrophils (10^9^/L)  Platelets (10^9^/L)  Glucose (mmol/L)  Sodium (mmol/L)  Potassium (mmol/L)  Urea (mmol/L)  Creatinine (mmol/L)  Magnesium (mmol/L)  Bilirubin (mmol/L)  PaO2/FiO_2_ Ratio | **N**  147  114  147  147  147  147  146  147  146  140  123 | **Median (IQR)**  0.51 (0.07-7.8)  0.23 (0.02-2.3)  14 (9-29)  9.4 (8-13)  135 (132-141)  3.3 (3.0-3.9)  7.9 (5.2-15.0)  93 (69-138)  0.80 (0.70-1.01)  23 (12-41)  169 (120-259) | **N**  93  73  93  93  92  92  92  93  92  89  73 | **Median (IQR)**  0.52 (0.07-6.6)  0.21 (0.02-1.7)  14 (9-26)  9.4 (8-12)  135 (132-139)  3.3 (2.9-3.8)  6.8 (3.9-12.3)  88 (56-115)  0.77 (0.67-1.01)  20 (12-32)  185 (135-301) | **N**  54  41  54  54  55  55  55  54  54  51  50 | **Median (IQR)**  0.39 (0.07-8.2)  0.23 (0.02-3.37)  14 (7-31)  9.4 (7-17)  136 (132-143)  3.4 (3.0-5.0)  12.0 (7.4-20)  118 (77-196)  0.86 (0.74-1.04)  31 (14-62)  155 (80-219) | ***P* Value**  0.78  0.43  0.805  0.75  0.36  0.12  <0.001  0.001  0.02  0.03  0.41 |

P value represents comparison between ICU survivors and non-survivors

Supplemental Table 3. Leukemia subtype in ICU survivors and non-survivors

|  | **ICU (N = 151)** | | | | | **1-Year Post ICU Admission (N = 150)** | | | | |
| --- | --- | --- | --- | --- | --- | --- | --- | --- | --- | --- |
|  | **Survivors**  **N=94** | | **Non-survivors**  **N=57** | | ***P* Value** | **Survivors**  **N=37** | | **Non-survivors**  **N=113** | | ***P* Value** |
| **Acute Myeloid Leukemia, N (%)** | 73 | 77.7% | 45 | 78.9% | 0.85 | 26 | 70.3% | 91 | 80.5% | 0.19 |
| M0 | 9 | 9.6% | 3 | 5.3% | 0.54 | 3 | 8.1% | 9 | 8.0% | 1 |
| M1 | 10 | 10.6% | 7 | 12.3% | 0.76 | 3 | 8.1% | 14 | 12.4% | 0.57 |
| M2 | 9 | 9.6% | 5 | 8.8% | 0.87 | 5 | 13.5% | 9 | 8.0% | 0.36 |
| M3 | 1 | 1.1% | 0 | 0% | 1 | 1 | 2.7% | 0 | 0% | 0.25 |
| M4 | 11 | 11.7% | 8 | 14.0% | 0.68 | 3 | 8.1% | 16 | 14.2% | 0.41 |
| M5 | 10 | 10.6% | 3 | 5.3% | 0.37 | 1 | 2.7% | 12 | 10.6% | 0.30 |
| Other | 23 | 24.5% | 19 | 33.3% | 0.34 | 10 | 27.0% | 31 | 27.4% | 0.96 |
| **Acute Lymphoblastic Leukemia, N (%)** | 21 | 22.3% | 12 | 21.1% | 0.85 | 11 | 29.7% | 22 | 19.5% | 0.19 |
| B-ALL | 18 | 19.1% | 10 | 17.5% | 0.93 | 9 | 24.3% | 17 | 15.0% | 0.25 |
| T-ALL | 4 | 4.3% | 1 | 1.8% | 0.65 | 2 | 5.4% | 3 | 2.7% | 0.60 |
| Mixed Phenotype | 0 | 0% | 2 | 3.5% | 0.14 | 0 | 0% | 2 | 1.8% | 1 |
